# The game between host antiviral innate immunity and immune evasion strategies of senecavirus A - A cell biological perspective

**DOI:** 10.3389/fimmu.2022.1107173

**Published:** 2022-12-22

**Authors:** Kuan Zhao, Shixia Zhang, Xiaona Liu, Xiaoran Guo, Zhaomeng Guo, Xiaozhan Zhang, Wanzhe Yuan

**Affiliations:** ^1^ College of Veterinary Medicine, Hebei Agricultural University, Baoding, China; ^2^ Hebei Veterinary Biotechnology Innovation Center, Hebei Agricultural University, Baoding, China; ^3^ College of Veterinary Medicine, Henan University of Animal Husbandry and Economy, Zhengzhou, China

**Keywords:** senecavirus A (SVA), antiviral innate immunity, ISGs, immune evasion, autophagy, stress granules

## Abstract

Innate immunity is the first line of the cellular host to defend against viral infection. Upon infection, viruses can be sensed by the cellular host’s pattern recognition receptors (PRRs), leading to the activation of the signaling cascade and the robust production of interferons (IFNs) to restrict the infection and replication of the viruses. However, numerous cunning viruses have evolved strategies to evade host innate immunity. The senecavirus A (SVA) is a newly identified member of the *Picornaviridae* family, causing severe vesicular or ulcerative lesions on the oral mucosa, snout, coronary bands, and hooves of pigs of different ages. During SVA infection, the cellular host will launch the innate immune response and various physiological processes to restrict SVA. In contrast, SVA has evolved several strategies to evade the porcine innate immune responses. This review focus on the underlying mechanisms employed by SVA to evade pattern recognition receptor signaling pathways, type I interferon (IFN-α/β) receptor (IFNAR) signaling pathway, interferon-stimulated genes (ISGs) and autophagy, and stress granules. Deciphering the antiviral immune evasion mechanisms by SVA will enhance our understanding of SVA’s pathogenesis and provide insights into developing antiviral strategies and improving vaccines.

## Introduction

Senecavirus A (SVA) is a single-strand positive-sense RNA virus belonging to the genus *Senecavirus* of the family *Picornaviridae* and contains only one serotype (1). It was first discovered incidentally in the cell culture medium as a contaminant in 2002 (2). It is not pathogenic in human and does not infect human cells (3), but it has been verified as an oncolytic virus that can propagate in tumor cells of human, so after the first isolation of SVA, it has been used as an oncolytic virotherapy candidate in humans (4–7). Since its first identification in 2002, SVA has been reported as a causative agent associated with sporadic cases of vesicular disease in pigs in the USA (8) and Canada (9). However, several continuous outbreaks of vesicular disease associated with SVA in swine farms were then reported in Canada (10), Thailand (11), Colombia (12), Vietnam (13), India (14), Brazil (15–17), China (18, 19) and USA (20) since 2014. The diseased swine are characterized by severe vesicular and/or ulcerative lesions on the oral mucosa, snout, coronary bands, and hooves, which are indistinguishable from the clinical symptoms caused by foot-and-mouth disease virus (FMDV), vesicular stomatitis virus (VSV) (21). Until now, the outbreaks of SVA caused considerable economic losses to the pig industry worldwide.

The genome of SVA is about 7.2 kb in length and contains a unique open reading frame (ORF) flanked by a 5’ untranslated region (UTR) and 3’ UTR, with a viral protein (VPg) covalently linked to 5’ end of the genome, and with a 3’ poly(A) tail. The SVA 5’ UTR also contains a hepatitis C virus (HCV)-like internal ribosome entry site (IRES), which recruits ribosomal subunits using a process independent of the cap-binding protein eukaryotic initiation factor (eIF) 4E. (22). The IRES of SVA was predicted to harbor domain II and domain III with pseudoknots which is essential for SVA translation (23, 24). Under the guidance of IRES, the ORF is translated into a single polyprotein and then processed by virus-encoded proteases into Leader protein and P1, P2, and P3 protein intermediate. P1 is further cleaved into four structural proteins, VP4, VP2, VP3, and VP1, responsible for binding to proteins such as the receptor antx-1 and inducing the neutralizing antibodies. The P2 and P3 are cleaved into nonstructural proteins 2A, 2B, 2C and 3A, 3B, 3C, and 3D, respectively (2), which are critical for the replication of SVA in the cells.

Innate immunity is the first line of host defense against invading pathogens that plays a vital role in restricting viral spread and replication. Upon viral infection, the released viral nucleotides are sensed by the host pattern recognition receptors (PRRs) in the cytoplasm or the nucleus and subsequently lead to the activation of a signaling cascade that ultimately results in the robust production of IFN, including IFN-α, IFN-β, and IFN-γ. IFNs bind to IFN receptors and then activate the Janus kinase (JAK)-signal transducer and activator of transcription (STAT) pathway leading to the transcriptional regulation of numerous IFN-regulated genes (ISGs), which exert numerous antiviral functions directly or indirectly (25–27). Although the hosts have developed highly efficient strategies to detect and control invading viruses to resist viral infection and spread, lots of viruses have evolved strategies to evade host defenses and thus effectively infect and replicate in host cells (28). As a cunning virus, increasing evidence suggests that SVA can evade the host’s antiviral effect in several ways for better infection and replication.

## Evasion of PRR signaling pathways

**Figure 1:**
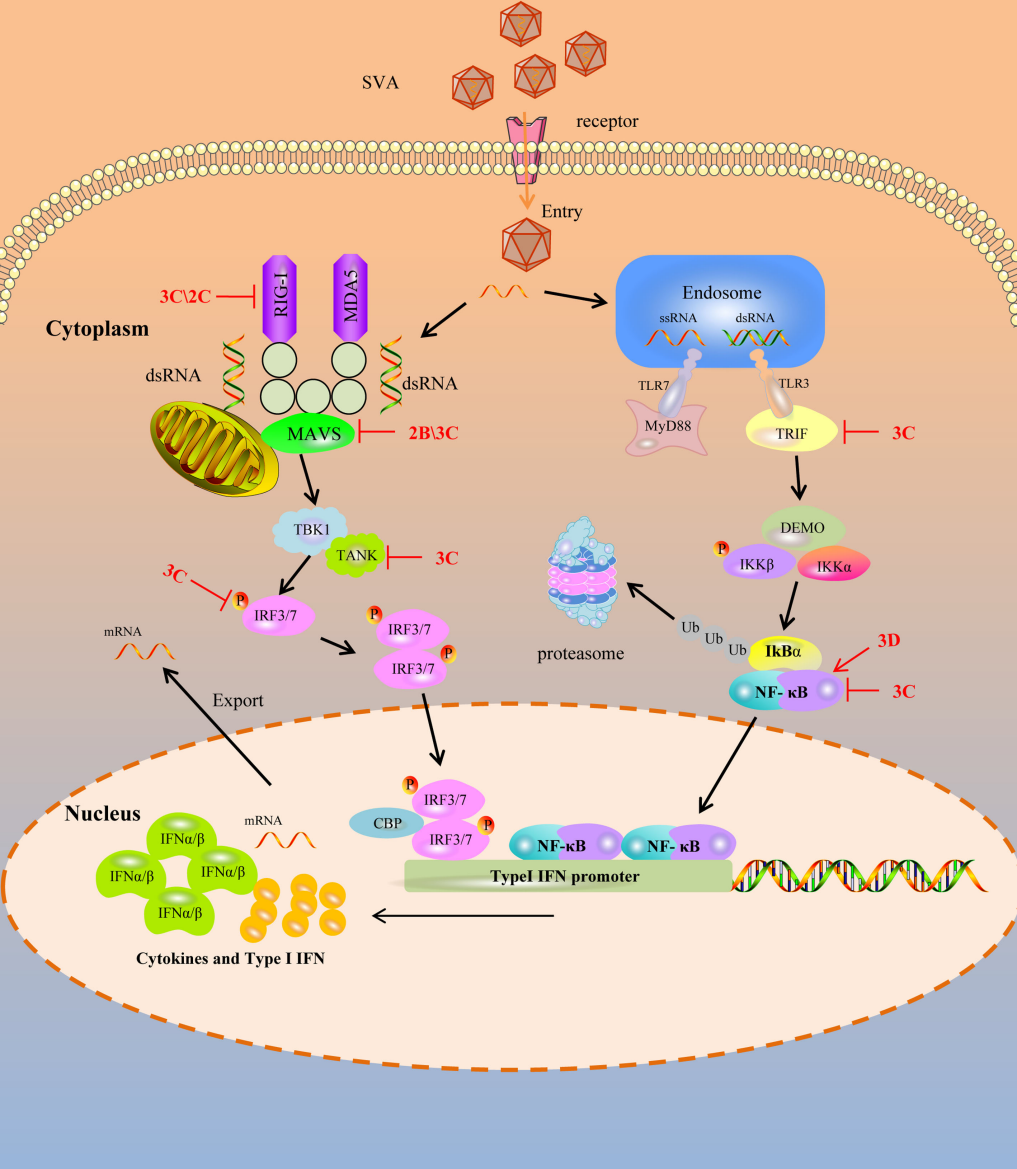
SVA escapes PRR mediated IFN-I signaling pathway. Cytoplasmic RNA sensors, such as TLR3, TLR7, RIG-1 and MDA-5, recognize SVA RNA in the cytosol and trigger the generation of IFN-I by transmitting a series of signals. SVA protein can target multiple steps in RLR-mediated IFN-I signaling pathway. The solid line represents the defined interaction between the adaptor and the SVA protein. The red arrow represents the promoting effect, and the red T-shaped symbol represents the inhibiting effect.

Pathogen-associated molecular patterns (PAMPs) are unique features in viruses that are recognized by PRRs to activate the innate immune response and proinflammatory cytokine responses during viral infection (25, 29). Upon viral infection, viral PAMPs are sensed by PRRs. Toll-like receptors (TLRs), RIG-I-like receptors (RLRs), and DNA sensors are mammals’ main PRRs that sense viral infection. TLRs are transmembrane proteins to recognize PAMPs derived from various microbes and initiate the transcription of inflammatory cytokines and IFNs. Among these TLRs, TLR3 recognizes the double-strand RNA, and TLR7 and TLR8 recognize the single-strand RNA during RNA viral infection (30–32). RLR9 recognizes DNA containing unmethylated CpG motifs in numerous viral and non-viral pathogens (33). Toll-interleukin 1 receptor domain-containing adapter inducing interferon-β (TRIF) is recruited by TLR3, and the myeloid differentiation factor 88 (MyD88) is recruited by TLR7 when they bind to the dsRNA and ssRNA respectively (34). However, the TLRs are limited in sensing viruses as they are only expressed in certain cell types (29). In contrast, RLRs, retinoic acid-inducible gene I (RIG-I), and melanoma differentiation-associated protein 5 (MDA5) were expressed in almost all of the cell types which can recognize the non-self RNA motif (29, 35). RIG-I recognizes short double-stranded (ds) RNA of viruses with 5′-phosphorylated blunt ends, whereas MDA5 binds long dsRNA molecules with no end specificity (36, 37). After RIG-I and MDA5 bind to RNA, it leads to the activation of a signaling cascade and recruits downstream ligand, mitochondrial antiviral signaling protein (MAVS, also known as IPS-1/VISA/Cardif), to activate IRF3 and NF-κB. RIG-I, MDA5 of porcine, two important sensors, interact with MAVS, the downstream adaptor, to activate the innate immune antiviral response during infection (38, 39). Of course, other different adaptors were also recruited. Still, the final result is to stimulate the two downstream kinases, tank-binding kinase 1 (TBK1) and inhibitor of κB kinase ϵ (IKKϵ), resulting in the phosphorylation and activation of transcription factors, including IFN regulatory factor 3 (IRF3), NF-κB, and AP-1 (40). These transcription factors combine to form transcription factor complexes and enter the nucleus, producing type I IFN. Type I IFN have a broad and diverse impact on the priming of expansion and maturation of adaptive immunity (41, 42). SVA has evolved complex strategies to evade type I IFN restriction, as illustrated in **Figure 1**, which is discussed in detail.

## Targeting RIG-I

RIG-I preferentially recognizes the short dsRNA characterized by blunt ends and a 5′ triphosphate moiety distinguishing host and viral dsRNA (35). RIG-I is under an auto-repressed state without dsRNA ligands. While upon the presentation of a viral dsRNA, the conformation of RIG-I is rearrangement to allow ATP binding to it, a necessary step for activating RIG-I (43). Once it is activated, the downstream adapter can be recruited and activated to induce the production of IFN. So, as a sensor, RIG-I can directly function as an effector in antiviral immunity. To complete viral replication and infection, picornaviruses have evolved strategies to antagonize the antiviral immunity of RIG-I by different methods, such as degrading and cleaving of RIG-I.

The L, 3C, and 2B proteins of the FMDV can degrade the RIG-I (44). EV71 3C protein targets RIG-I to block subsequent recruitment of adaptor molecule MAVS and inhibit consequent nuclear translocation of IRF3, and it also inhibits the ubiquitination of RIG-I to block IFN production (45, 46). Besides, the 3C protein of poliovirus and encephalomyocarditis virus (EMCV) is responsible for the cleavage of RIG-I (47). SVA, as a picornavirus, can evade the host’s innate immunity. Overexpressing RIG-I can significantly restrict the replication of SVA, while RIG-I was degraded in SVA-infected cells, with 2C and 3C playing essential roles in this process. Although 3C can interact with RIG-I, 2C cannot. They both significantly reduced Sev or RIG-I-induced IFN-β production. Moreover, 2C and 3C-induced RIG-I degradation depends on the caspase signaling pathway (48). So, the antiviral immunity induced by RIG-I against SVA is weakened by the 2C and 3C of SVA.

## Targeting MAVS

The mitochondrial antiviral signaling protein (MAVS) is an important adaptor protein in host anti-RNA virus immunity. It contains an N-terminal CARD domain that interacts with the tandem CARD domains of RIG-I and the C-terminal transmembrane domain that localizes itself to the mitochondrial outer membrane. It mediates the activation of NF-κB and IRFs and induces the production of IFN (49). During viral infection, weakening or blocking the MAVS function will play a multiplier effect in resisting the host’s antiviral immunity.

SVA has evolved the ability to suppress the host’s innate immune responses to benefit its replication by blocking type I IFN production and ISG expression. Previous studies showed that the 2B protein of SVA can decrease the expression of both exogenous and endogenous MAVS in dose-dependent manners. In contrast, the decrease of MAVS was not associated with the formation of insoluble fractions and the cleaved process. 2B protien of SVA degraded the MAVS by colocalized and interacting with MAVS depending on caspase-9 and caspase-3. In addition, the 1-48 and 100-128aa regions of 2B were essential for inhibiting the type I IFN production (50). Besides, the 3C protein of SVA can interact with MAVS, and the cleavage of the MAVS depends on its protease activity. The cellular apoptosis and degradation process impairs the cleavage of MAVS by the 3C protein. The fragments of MAVS cleavaged by 3C protein lost their activity to induce IFN production (51). So, for MAVS, an important adapter protein, the cunning SVA blocks the host antiviral innate immunity by weakening MAVS biological functions through its 2B and 3C protein.

## Targeting IFN regulatory factor

IRF3 and IRF7 are important molecules in virus-mediated induction of type I IFN production. Normally, they remain in the cytoplasm without phosphorylation. During viral infection, the activation of innate immunity could induce IRF3 and IRF7 to undergo phosphorylation, dimerization, and translocation into the nucleus, leading to the expression of IFN which then induce the production of ISGs (52, 53). For this important effect of innate immunity, SVA has evolved strategies to antagonize this antiviral process. Previous researchers found that the 3C protein of SVA inhibited the expression of IRF3 and IRF7 depending on its protease activity. If the catalytic box of the 3C protein was mutated, it failed to mediate the reduction of IRF3 and IRF7. Moreover, it can interact with IRF3 and IRF7 and induce a reduction in the phosphorylation of IRF3 and IRF7 by the protease activity to suppress the production of IFN (54). While for FMDV, another member of picornavirus, reduces the expression of IRF3 and IRF7 by the L protein depending on the protease activity (55). So the 3C protein of SVA functions similarly to the FMDV L protein in antagonizing the innate immune response.

## Targeting TRIF and TANK

Toll-like receptors, including TLR3, TLR7, TLR8, TLR9, RIG-I, and MDA5, are the main PRRs to recognize the virus RNA and induce the production of IFN. In the TLR3 signaling pathway, TRIF regulates TLR3-mediated IRF3 and NF-κB activation (56). Besides, tumor necrosis factor receptor-associated factor (TRAF) family member-associated NF-κB activator (TANK) is critical in regulating RLR- and TLR-mediated interferon production. TANK regulates the TBK1-IKK-mediated IFN antiviral response by interacting with several signal molecules, such as MAVS, TRIF, TBK1, and IRF3 (57). Although the host has evolved various antiviral strategies to defend against viral infection, the virus can still complete its replication and infection naturally, so there must be ways to antagonize the antiviral effect of the host. For SVA, during its infection, it failed to trigger host IFN production and the 3C protein showed an extremely inhibitory effect. Further investigation showed that the inhibition of IFN production by 3C depends on the cleavage of TRIF, TANK, and MAVS (51). Furthermore, the cleavage of TRIF and TANK depends on the activity of 3C by interacting with them. TRIF and TANK cleavage fragments lost their functions to induce IFN production. So the SVA antagonizes the host antiviral innate immunity by cleaving TRIF, MAVS and TANK molecular which are crucial for the TLR3 mediated and RLR mediated signaling pathway (51).

## Targeting NF-κB

NF-κB is a ubiquitous transcription factor that regulates innate immunity and inflammatory responses. The NF-κB signaling pathway plays an important role in the virus life cycle. The NF-κB pathway is usually activated by RIG-I/MAVS or cGAS/STING signaling cascades, begins with the cellular PRRs recognizing the PAMPs, especially virus RNA, and then delivers the signaling cascade, which induces the transcription of interferon related genes and then restricting the replication of viruses (58). The NF-κB signaling module consists of five NF-κB monomers (RelA/p65, RelB, cRel, NF-κB1 p50, and NF-κB2 p52), which can dimerize to form up to 15 unique transcription factors (59). NF-κB-p65 is a key NF-κB subunits directly responsible for transactivating NF-κB target genes. In the early stage of SVA infection, the host activates NF-κB by recognizing SVA RNA and then induces a signaling cascade that causes transcriptional expression of downstream molecules to exert antiviral effects (60). However, at the late stage of infection, the NF-κB-p65 could be cleaved by the 3C protein of SVA. While, further studies indicated that the cleavage of NF-κB-p65 is not the direct action of the 3C protein but mediated by caspases. Besides, SVA infection can induce the apoptosis of host cells to promote the replication of itself. Interestingly, NF-κB-p65 prevents the apoptosis induced by SVA, so at the late-stage infection, the cleavage of NF-κB-p65 and induction of host cell apoptosis may be critical for SVA replication and release from infected cells (61, 62). Moreover, the 3D protein of SVA could promote the activation of NF-κB by binding IKKα and IKKβ, which further upregulates the NLRP3 and pro-IL-1β transcription. Then, the N-terminal of 3D promotes the assembly of the NLRP3 inflammatory complex to induce IL-1β production by binding to the NACHT domain of NLRP3 (63), which may be another way for the cunning SVA to evade the innate immunity strategy.

## The game between the intrinsic antiviral proteins and SVA

**Figure 2:**
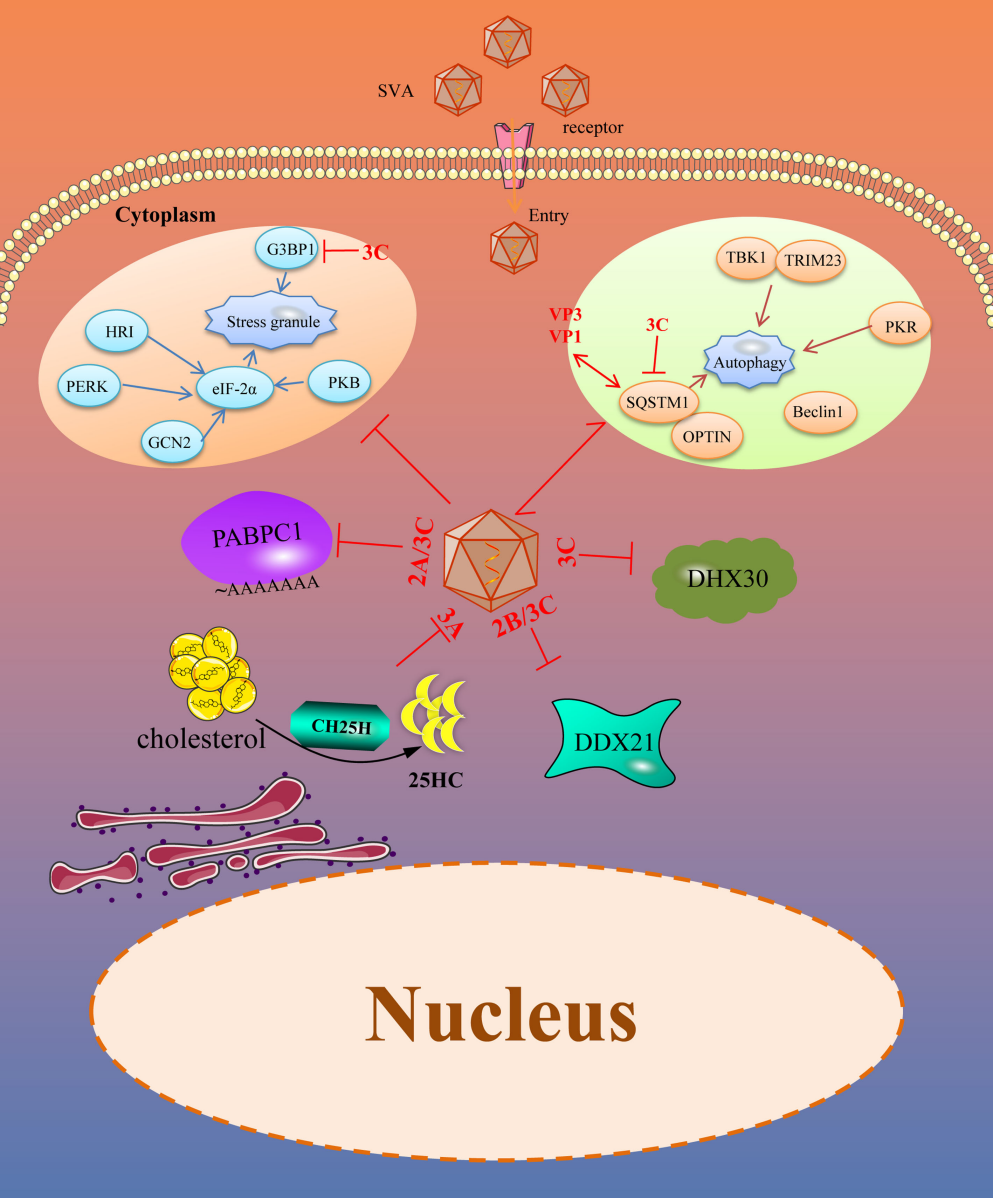
SVA evades intrinsic antiviral proteins as well as autophagy and stress granules (SGs). Viral proteins of SVA engage multiple strategies to evade the restriction of intrinsic antiviral proteins. In addition, SVA infection can trigger some other immune responses, such as SGs and autophagy, which helps to limit viral infection. However, these immune responses are also controlled by viral proteins. The solid line represents the defined interaction between the adapter and the SVA protein. The red two-way arrow represents interaction, and the red T-shaped symbol represents the inhibiting effect.

Many intrinsic antiviral proteins can inhibit the replication of SVA, and at the same time, SVA has evolved multiple ways to antagonize thegse proteins. The evasion of ISGs and intrinsic antiviral proteins by SVA is illustrated in **Figure 2**.

## DDX21

DEAD (Asp-Glu-Ala-Asp)-box RNA helicases (DDXs) are the largest family of evolutionarily conserved RNA helicases that are involved in a broad array of host processes, especially in antiviral immunity (64, 65). DDX21, a member of the DDX family, possesses all the signature motifs required for DEAD-helicase function and contains atypical FRGQR repeats in its C-terminus. Furthermore, growing evidences suggest that DDX21 plays an important role in regulating host antiviral immunity against picornaviruses. DDX21 regulated the replication of FMDV by increasing IFN-β and IL-8 production in FMDV infected cells. It also co-precipitates with FMDV IRES and restricts viral IRES-dependent translation and replication (66). Our previous study suggested that DDX21 restricted the replication of SVA in PK15 and BHK-21 cells. Overexpression of DDX21 in the cells suppressed the replication of SVA and knocking down the expression of DDX21 promoted the replication of SVA. In contrast, SVA can effectively replicate in the natural infection condition. Therefore, SVA inevitable could evade the antiviral effect of DDX21. Our further investigation revealed that the expression level of DDX21 gradually decreased with the prolongation of infection time. So, SVA evades the antiviral activity of DDX21 mainly dependent on decreasing its expression. 2B and 3C proteins of SVA were critical for the degradation of DDX21, which depends on the caspase pathway. Moreover, when the activity sites of the 3C protein were mutated, the protease activity was lost. The mutated 3C failed to induce the degradation of DDX21. All of these suggested that the protease activity of 3C protein was necessary for the degradation of DDX21, which contributed to SVA evading the antiviral effect of DDX21 (67).

## DHX30

Another RNA helicase, DExH-box helicases (DExH), can act as a sensor molecule to regulate antiviral innate immunity and exert direct antiviral effects by targeting viral proteins or RNA (68). DHX30, a multi-role member of DExH, is involved in the biosynthesis of mitochondrial ribosomes (69) and can be recruited by Zinc-finger antiviral protein (ZAP) through interacting with each other and then increase the antiviral effects of ZAP (70). It can inhibit the replication of numerous viruses, such as HIV-1 and influenza A virus, through different molecular mechanisms (71, 72). Researchers have shown that overexpression of DHX30 inhibits the replication of SVA, and downregulated DHX30 promotes the replication of SVA at the early stage of the life cycle, depending on its helicase activity. For SVA, to antagonize the anti-SVA effects of DHX30, during the infection of SVA, the 3C protein of SVA interacts with DHX30 and cleaves DHX30 at the Q220 site. The SVA 3C protein cleaves DHX30 through its protease activity and independent cellular caspases. Though 3C-mediated DHX30 cleavage products still bound SVA RNA, they lost the ability to inhibit virus replication. The researchers speculated that the cleavage products lost the helicase activity, so they could not exert an antiviral effect. Nevertheless, the reason for this need further investigation (73).

## CH25H

Cholesterol-25-hydroxylase (CH25H) is an ISG induced by IFN. As a member of ISG protein, it can convert cholesterol to 25-hydroxy cholesterol (25HC) (74). 25HC is a soluble factor that suppresses sterol synthesis by regulating sterol-responsive element binding proteins (SREBP) and nuclear receptors, which is reported to inhibit the stage of viral abortion and entry (75, 76). Researchers have demonstrated that CH25H and 25HC can suppress many viruses infection progresses, such as Zika virus (ZIKV), Porcine epidemic diarrhea virus (PEDV), Pseudorabies virus (PRV), EMCV, Porcine reproductive and respiratory syndrome virus (PRRSV) and Herpes simplex virus type 1 (HSV-1) (77–82). In addition, CH25H and 25HC can also inhibit SVA. Overexpression of CH25H inhibits SVA replication. On the contrary, knockdown or knockout of the endogenous CH25H promotes SVA infection. Further, 25HC exerts its antiviral effect by inhibiting virus replication and attachment. Interestingly, the CH25H-M (CH25H mutant) lacking hydroxylase activity still retains its antiviral properties through selectively interaction and degrade SVA 3A protein *via* the ubiquitin-proteasome manner. The antiviral effect of CH25H was dependent and independent of its enzymatic activity (83). For 25HC, it exerts its antiviral effect in the entire life cycle of SVA, especially in the adsorption process of SVA (84). Different viruses have developed strategies to antagonize the antiviral effect of CH25H and 25HC. PRRSV nsp1β and nsp11 can degrade the CH25H by lysosomal pathway. Moreover, the E protein of PRRSV degrades the CH25H by the ubiquitin-proteasome pathway (85, 86). For SVA, CH25H expression was downregulated during SVA infection and the degradation of CH25H becomes more serious with the prolongation of infection, which maybe a strategy for SVA to antagonize the antiviral effect of CH25H (83). In addtion to, from the results of previous study we found that after CH25H-M was co-transfected with SVA protein, CH25H-M could be degraded by VP4, 2B and 3C protein of SVA to a certain extent (84). All of these revealed that the SVA maybe escape the antiviral effects of CH25H by degraded it with some viral proteins, but it needs further investigation.

## PABPC1

The poly(A) binding protein cytoplasmic 1 (PABPC1) is a poly(A) binding protein which consists of a globular domain, four non-identical RNA-recognition motifs (RRMs) and a proline-rich C-terminal domain (87, 88). In the cells, the poly(A) binding protein, mRNA, and eukaryotic translation initiation factor 4 gamma (eIF4G) interacted with each other to constitute a complex that initiating the translation and mRNA circularization (89). Studies have confirmed that PABPC1 is an antiviral protein against SVA. During SVA infection, the PABPC1 was cleaved at residue 437 mediated by the 3C protein through its protease activity which was similar to the EMCV (90). As PABPC1 is critical for protein synthesis and the PABPC1 cleaved by 3C protein will decrease the protein synthesis rates. SVA infection can inhibit the cellular protein synthesis rates over time and interfer with the cell defense system. Besides SVA, the NSP3A protein of rotavirus can bind to eIF4G to transfer PABPC1 from the translation complex (91). The 2A and 3C proteases of picornavirus can inactivate PABPC1 by cleaving the N-terminal of PABPC1 resulting in it cannot bind to eIF4G, thus affecting the normal translation of the host (90, 92). Poliovirus 3C protease cleaves poly(A) binding protein and eIF4G to inhibit host cell translation (93). So, clearing poly(A) binding protein may be a common method for picornavirus to antagonize the antiviral effect of PABPC1.

## SVA evades autophagy and stress granules

Viruses are acellular organisms whose life cycle must depend on the host cell enzyme and translation system. The virus is only composed of proteins and nucleic acids. During the viral infection process, the proteins or nucleic acids act as foreign substances that may stimulate the stress responses of the host cells, including autophagy, stress granules, apoptosis, and pyroptosis. While we only discuss autophagy and stress granules antagonized by SVA (**Figure 2**).

## Autophagy

Autophagy is a conserved cellular process important for cell survival and homeostasis. Autophagy enables cells to recycle nutrients and remodel and dispose of unwanted cytoplasmic constituents, critical for protecting the host cells from pathogenic infections (94). Recently, many studies have focused on the antiviral effect of autophagy and found that autophagy is an effective defense strategy against a wide variety of invading viruses (95, 96). However, viruses including Newcastle disease virus (NDV), PRRSV, EMCV, FMDV, PEDV have developed multiple strategies to antagonize the host autophagy process for their benefit (97–101). SVA infection can induce autophagy in different cells by detecting autophagosome formation, GFP-LC3 puncta, and accumulation of LC3-II proteins. However, autophagy suppresses or promotes SVA replication in a species-specific manner, restricting SVA replication in human cells and promoting SVA replication in pig cells (102, 103). Sequestosome 1 (SQSTM1), a selective autophagy receptor, interacts with VP1 and VP3 of SVA and targets them to phagophores for degradation to inhibit viral replication. To counteract this, the 3C protein of SVA targets the receptor SQSTM1 for cleavage at glutamic acid 355, glutamine 392, and glutamine 395 and abolishes its capacity to mediate selective autophagy. Besides, the cleavage products of SQSTM1 mediated by 3C protein lost the ability to inhibit viral propagation (103). In addition, the 2AB protein of SVA interacts with MARCHF8/MARCH8 and LC3 to antagonize the antiviral effect of autophagy. MARCHF8 can combine with MAVS to form a complex and stimulate the IFN-I signaling. The interaction of MARCHF8 and 2AB prevents this combination to deactivate IFN-I signaling. LC3 is also degraded by 2AB and inhibits autophagy (104). So, SVA can evade the autophagy to promote viral replication, mainly dependent on viral 3C and 2AB protein.

## Stress granules

Stress granules (SG) are mRNA storage sites that regulate mRNA translation, localization, and degradation. SG responds to various environmental stress and viral infection and is one of the pathways by which host cells respond to pathogenic infection. Viral infection can cause cellular stress and regulate gene expression by influencing mRNA translation, localization, and degradation (105). There is a close relationship between SG and viral infection replication. Four different SG formation patterns exist during viral infection, including no SG formation, stable SG formation, transient SG formation, and alternate SG formation. Several studies have confirmed that many viruses can induce stable SG formation, such as the PRRSV and NDV (106, 107). As the inhibitory effect of SGs on numerous virus replication, different viruses have evolved unique strategies to prevent SG formation and promote efficient viral propagation. SVA infection induces transient SG formation *via* a PKR-eIF2a-dependent manner at the early stage of infection, and this transient SG is not related to the replication effect of SVA. Besides, Ras-GTPase-activating protein (SH3 domain) binding protein 1 (G3BP1) is a stress granule-resident protein and G3BP1 induced SGs are related to the activation of innate immune responses through NF-κB and JNK. Researchers have found that SVA infection inhibits the SG formation by 3C protein depending on its protease activity at the late stage of infection. In addition to, the 3C protein also disrupts eIF4GI-G3BP1 interaction, which blocks the SG formation (108). However, the significance of SVA blocking SG formation needs further studies.

## Conclusions and discussions

The host’s innate immune response is the first line of defense against infection by pathogenic microorganisms. Viruses have evolved various strategies to evade the antiviral effect of the host for better proliferation. SVA causes swine vesicular disease, which is clinically indistinguishable from Foot-and-mouth disease and Vesicular stomatitis. It is associated with an increased number of outbreaks in pigs in several countries. This review summarizes the strategies of SVA to counteract the antiviral innate immune responses. Many proteins of SVA are involved in this process, especially the nonstructural protein, including 3C and 2C. 3C or 2C protein can cleave and degrade the key components of PRR signaling pathways to suppress the production of IFN, including RIG-I, MAVS, IRF3, IRF7, TRIF, and TANK. Besides, some ISGs and antiviral proteins are cleaved by nonstructural proteins, especially 2B, 2C, and 3C proteins. Most of the cleavages by 3C or 2C proteins depend on their protease activity. These nonstructural proteins might be an excellent target for developing antiviral drugs.

Currently, there is no research on SVA antagonizing the JAK-STAT signaling pathway, which is the main signaling pathway for ISG production. Therefore, it is not explained in the review. However, other picornaviruses, such as FMDV, and EMCV, could utilize VP3 and 3C proteins targeting JAK1, JAK2, IRF9, and STAT to evade the antiviral effect (109). It is necessary to be clarified how SVA antagonizes the JAK-STAT signaling pathway and the VP3 and 3C proteins of SVA might be the focus of the research in the future. Besides, for pyroptosis and apoptosis, SVA induces but does not inhibit these responses. And, SVA-induced apoptosis and pyroptosis contribute to the replication of SVA in tumor cells and promote an oncolytic effect (110, 111).

A detailed understanding and careful examination of the mechanisms of how SVA evades the host immune system will help develop new antiviral drugs for the treatment of SVA, as well as highly efficient vaccines for the prevention of related diseases.

## Author contributions

KZ conceptualized the idea and wrote the manuscript. SXZ, XNL, XRG and ZMG generated the figures. WZY and XZZ made constructive comments to the review. All authors contributed to the article and approved the submitted version.
